# Links between Air Pollution and Aortic Diseases: Current Evidence for Future Prevention and Treatment

**DOI:** 10.7150/ijms.124106

**Published:** 2026-01-19

**Authors:** Chayatorn Chansakaow, Poon Apichartpiyakul, Siriporn C Chattipakorn, Nipon Chattipakorn

**Affiliations:** 1 Vascular Surgery Unit, Department of Surgery, Faculty of Medicine, Chiang Mai University, Chiang Mai 50200, Thailand.; 2 Cardiac Electrophysiology Research and Training Center, Faculty of Medicine, Chiang Mai University, Chiang Mai 50200, Thailand.; 3 Center of Excellence in Cardiac Electrophysiology Research, Chiang Mai University, Chiang Mai 50200, Thailand.; 4 Department of Oral Biology and Diagnostic Sciences, Faculty of Dentistry, Chiang Mai University, Chiang Mai 50200, Thailand.; 5 The Academy of Science, The Royal Thai Society of Thailand, Bangkok, Thailand.

**Keywords:** aorta, aortic aneurysm, air pollution, oxidative stress, inflammation

## Abstract

This review examines the evidence linking air pollution, particularly fine particulate matter (PM2.5), to aortic diseases such as atherosclerosis, aneurysms, and dissections. Air pollution is a significant environmental risk factor for cardiovascular disease, and understanding its impact on the aorta is crucial for developing prevention strategies. We performed a comprehensive literature search of PubMed for articles published between December 2007 and May 2024, including *in vivo*, *in vitro*, and clinical studies that investigate the effects of air pollution on aortic pathophysiology. Findings indicate that exposure to PM2.5 and diesel exhaust particles accelerates aortic atherosclerosis, aneurysm formation, and dissection through mechanisms involving oxidative stress, inflammation, endothelial dysfunction, and vascular remodelling, with heightened effects in genetically predisposed models and high-fat diets. *In vitro* studies reveal that particles can cause cytotoxicity in human aortic endothelial cells, characterized by reduced nitric oxide production and cellular damage. Clinical data are mixed but suggest associations between air pollution and increased aortic calcification, arterial stiffness, and altered hemodynamics. Overall, air pollution influences the development and progression of aortic diseases via multiple biological pathways, emphasizing the need for further research to define dose-response relationships, identify molecular targets, and implement environmental interventions to reduce disease burden and protect public health.

## Introduction

Air pollution has emerged as one of the most pressing environmental health concerns of the 21^st^ century. As urbanization and industrialization continue to accelerate, the quality of the air we breathe is increasingly compromised by pollutants such as particulate matter (PM), nitrogen oxides (NOx), sulfur dioxide (SO_2_), and ozone (O_3_). Among these, fine particulate matter (PM2.5) has been found to be significantly important due to its ability to penetrate deep into the lungs and enter the bloodstream, leading to a myriad of adverse health effects. While the respiratory impact of air pollution is well-documented, recent research has begun to elucidate its profound effects on cardiovascular health.^1^

The aorta, the body's largest artery is responsible for delivering oxygenated blood from the heart to all organ systems and other important tissues. Aortic diseases include various conditions, ranging from genetic disorders to lifestyle factors such as high blood pressure and atherosclerosis. These conditions can result in serious complications such as aortic aneurysms, dissections, and other potentially fatal events. The global burden of aortic disease is significant, with a high mortality rate that underscores the need for urgent attention. Specifically, the worldwide mortality rate related to aortic disease, including thoracic aortic aneurysm (TAA), abdominal aortic aneurysm (AAA), and acute aortic dissection (AAD) was 2.78 per 100,000 individuals in 2010. 2 More recent Global Burden of Disease analyses indicate that aortic aneurysm remains a significant contributor to global mortality, particularly in aging populations.^3^

Epidemiological studies have revealed a troubling correlation between exposure to air pollution and increased incidence of vascular diseases. The mechanisms underlying this association are thought to involve oxidative stress, inflammation, and endothelial dysfunction which are all exacerbated by pollutants.^4^, ^5^ These pollutants contribute to the pathogenesis of atherosclerosis, a primary driver of many vascular diseases, particularly those affecting the aorta, leading to various aortic conditions.

Considering these challenges, this review aims to explore the intricate link between air pollution and aortic disease. By comprehensively summarizing the latest scientific evidence, we seek to highlight our current understanding pertinent to the pathophysiological mechanisms underlying these adverse effects of pollutants on aortic disease. Understanding and addressing these connections is crucial for future development of effective strategies that can improve cardiovascular outcomes in aortic disease patients and enhance overall public health resilience amid the rapid increase in pollution globally.

## Methods

A comprehensive literature search was conducted in PubMed for articles published between December 2007 and October 2025. The search terms included “aorta,” “aortic aneurysm,” “air pollution,” and “particulate matter.” Only original research and review articles published in English were included. Relevant *in vivo*, *in vitro*, and clinical studies investigating the effects of air pollution on aortic pathophysiology were selected for review.

### Air Pollutants induce aortic atherosclerosis and aortic aneurysm: Evidence from *in vivo* studies

Compelling evidence from various *in vivo* models demonstrates that exposure to particulate air pollutants, especially fine PM2.5 and diesel exhaust particles (DEP), can initiate and exacerbate the development of aortic diseases for example atherosclerosis, aortic aneurysms, and aortic dissections. A comprehensive summary of reports from *in vivo* studies are shown in Table 1A-1C. In mouse models PM2.5 exposure induced the progression of aortic atherosclerosis, as evidenced by increased lesion areas, lipid deposition, inflammatory markers, and upregulation of atherogenic proteins like CD36.^6^-^8^ The severity of atherosclerosis was correlated with PM2.5 concentration and exposure duration. It was also found that exposure to PM2.5 in mice promoted formation of an abdominal aortic aneurysm and aortic dissection.^6^, ^9^ Similarly, exposure to PM10 in rabbits accelerated atherosclerosis by increasing monocyte recruitment into plaques and inducing systemic inflammation.^10^, ^11^ Exposure to diesel exhaust particles also induced pro-atherosclerotic and oxidative stress responses in the aorta of ApoE^-/-^ mice, with effects such as increased macrophage infiltration, collagen deposition, and upregulation of factors involved in vascular remodeling. Even short-term inhalation (several days up to 7 days) of secondary organic aerosols induced vascular inflammation in this model.^12^-^15^ Interestingly, these animal studies implicate particulate air pollution, especially PM2.5 and diesel exhaust particles, in exacerbating the pathogenesis of aortic atherosclerosis and aortic aneurysms/dissections through mechanisms involving oxidative stress, inflammation, endothelial dysfunction and pathological vascular remodeling.

According to current evidence, PM2.5 (fine particulate matter) appears to have the most significant and well-documented effects on aortic pathologies. Several studies demonstrate its impact on promoting atherosclerosis, increasing aortic aneurysm formation, enhancing aortic dissection, inducing inflammation and oxidative stress in perivascular adipose tissue (PVAT), impairing endothelial function, and promoting vascular insulin resistance.^6^, ^7^, ^9^ Despite these important findings, knowledge gaps still exist. Most studies focus on short- to medium-term exposures (ranging from several days up to approximately 3-6 months), and there is a lack of data on the effects of lifelong exposure. More detailed investigations into dose-response relationships could help establish exposure thresholds. Additionally, studies on whether the observed effects are reversible after cessation of exposure are limited. Addressing these gaps is essential for a comprehensive understanding of the long-term impacts of exposure to PM2.5.

### Air pollutants induce aortic atherosclerosis and aortic aneurysm: evidence from *in vivo* reports through the comparison of the manipulation of genetic and/or dietary factors

Genetic risk factors such as ApoE/LDLR deficiency and environmental risk factors such as high-fat or high-cholesterol diets have been demonstrated to modulate the effects of air pollutant exposure on aortic atherosclerosis and aneurysm formation. A comprehensive summary of these reports is shown in Table 2A-2B. Exposure to PM2.5 was shown to exacerbate atherosclerotic plaque formation and inflammation to a greater extent in ApoE^-/-^ mice fed a high-fat diet, compared to those fed on a normal diet.^16^, ^17^ This finding indicates that the pro-atherosclerotic effects of PM2.5 are amplified in the setting of dyslipidemia. Similarly, exposure to mixed air pollutants was shown to cause greater increases in atherosclerosis plaque thickness in hyperlipidemic LDLR^-/-^ mice, in comparison to wild-type mice.^18^ Interestingly, the antioxidant N-acetylcysteine (NAC) effectively attenuated PM-induced atherosclerosis in LDLR^-/-^ mice, suggesting oxidative stress as a key pathogenic mechanism.^19^ These findings also highlight the synergistic interactions between genetic susceptibilities such as ApoE/LDLR deficiency, environmental risk factors such as high-fat diet, and exposure to particulate air pollution in promoting the initiation and progression of aortic atherosclerosis. Moreover, these reports provide evidence that exposure to particulate matter could amplify atherogenesis in the milieu of hyperlipidemia, potentially via oxidative stress pathways.

### Air pollutants induce aortic disease: evidence from *in vitro* studies

The mechanistic insights into how particulate air pollutants can directly impair the function and viability of human aortic endothelial cells, thereby disrupting the anti-atherosclerotic properties of the endothelium, were demonstrated using *in vitro* models. A summary of these reports is shown in Table 3. It has been shown that exposure to ultrafine particles (diameter < 200 nm) could impair the production of nitric oxide by human aortic endothelial cells via mechanisms involving endothelial nitric oxide synthase dysfunction and S-glutathionylation.^20^ Nitric oxide is a key endothelium-derived vasodilator and anti-atherogenic factor, therefore the impairment of nitric oxide production caused by particulate pollutants could promote endothelial dysfunction and accelerate atherosclerosis development. Exposure to diesel exhaust particles was also shown to decrease the viability of human aortic endothelial cells in a dose-dependent manner.^21^ It also altered cytoskeletal structures, induced plasma membrane damage, and decreased mitochondrial membrane potential. These cellular perturbations could compromise the barrier and signaling functions of the endothelium that normally protect against atherogenesis.

Taken together, these *in vitro* studies elucidate some of the direct detrimental effects of particulate pollutants such as ultrafine particles and diesel exhaust particles on aortic endothelial cells at molecular and structural levels. Such endothelial injury and dysfunction can significantly contribute to the pro-atherogenic effects observed in the *in vivo* studies. Recent evidence indicates that aqueous extracts of PM₂.₅ can trigger pro-inflammatory macrophage polarization (M1 phenotype), promote intracellular lipid accumulation, and upregulate stress-response proteins such as HSP70. These findings support the notion that particulate pollution may foster vascular inflammation through macrophage activation, thereby accelerating atherogenic processes.^22^ Consistently, Caceres et al. (2024) demonstrated that PM_2.5_ exposure primes macrophages to activate the NLRP3 inflammasome and release IL-1β, highlighting a key inflammatory pathway linking particulate matter to vascular injury. 23

However, it is important to note that only a few *in vitro* studies have been conducted so far, which limits the understanding of the full spectrum of potential mechanisms. Moreover, other pollutants, such as polycyclic aromatic hydrocarbons, and secondary organic aerosols, may also have significant effects on endothelial cells. Further research is needed to explore these additional pollutants and their potential mechanisms of action, which could provide a more comprehensive understanding of how air pollution contributes to endothelial injury or dysfunction.

### Air pollutants induce aortic disease: evidence from clinical studies

An increasing number of studies have investigated the possible connection between exposure to air pollutants, especially in relation to particulate matter (PM), and the development and progression of subclinical atherosclerosis. A comprehensive summary of these clinical reports is shown in Table 4. The prospective panel study in Beijing demonstrated a significant association between elevated levels of PM2.5, PM10, black carbon, SO_2_ and NOx and increased central aortic systolic pressure, carotid artery stiffness, and augmentation index, suggesting adverse hemodynamic effects on the cardiovascular system.^24^ Similarly, a German prospective cohort study found that higher PM_2.5_ exposure was associated with a greater burden of thoracic aortic calcification (TAC), a marker of subclinical atherosclerosis.^25^

Unlike the findings from those prospective investigations, several conflicting findings have been reported from other retrospective and prospective studies. A retrospective cross-sectional study in US metropolitan areas did not find an association between PM_2.5_ levels and abdominal aortic calcification in middle-aged and older adults.^26^ Likewise, a German retrospective cohort study and a prospective cohort study in Northeastern USA did not find significant associations between traffic-related air pollution or PM_2.5_ exposure and the development, progression, or extent of TAC or abdominal aortic calcification.^27^, ^28^

Since there are several limitations observed in the clinical reports, these divergent results from clinical investigations could be attributed to differences in study design, population characteristics, exposure assessment methods, and other factors. A further significant limitation is the lack of a clear mechanism that explains the link between particulate matter (PM) exposure and vascular conditions. Without a comprehensive understanding of the mechanisms, it is challenging to determine causality and develop targeted interventions. Further research is warranted to elucidate the potential mechanisms and clarify the role of exposure to air pollution in the pathogenesis of subclinical atherosclerosis, which could have important implications for cardiovascular disease prevention and public health policies.

## Future Perspectives and Research Directions

Despite the limited number of studies on the effects of air pollutants on aortic disease, the current findings from those reports ranging from *in vitro* to *in vivo* and in clinical investigations highlight several important future research directions to better understand and mitigate the detrimental effects of particulate air pollutants on aortic diseases including atherosclerosis, aortic dissection and aortic aneurysms. Further dose-response studies in both animal models and human cohorts are needed to define safe exposure thresholds and characterize the risk associated with long-term exposure to particulate pollution. Mechanistic studies elucidating the specific cellular and molecular pathways disrupted by distinct pollutants such as PM_2.5_ and diesel exhaust particles could identify novel therapeutic targets or preventive strategies. Development of simple biomarkers associated with particulate exposure would enable monitoring of high-risk patients in clinical practice. Moreover, the role of dietary, nutritional factors and medication in modulating pollutant toxicity warrants deeper investigation, as certain nutrient deficiencies or supplements may exacerbate or mitigate pollutant-induced arterial injury. Comprehensive studies tracking the full spectrum from environmental exposure to disease pathogenesis and progression could inform important preventive and therapeutic strategies tailored to an individual's exposure profile. Ultimately, a multi-pronged research approach integrating environmental monitoring, epidemiological studies, basic mechanistic investigations, and clinical translation will be crucial to combat the growing public health challenge posed by the deleterious cardiovascular effects of ubiquitous particulate air pollutants.

## Conclusion

**Figure 1:**
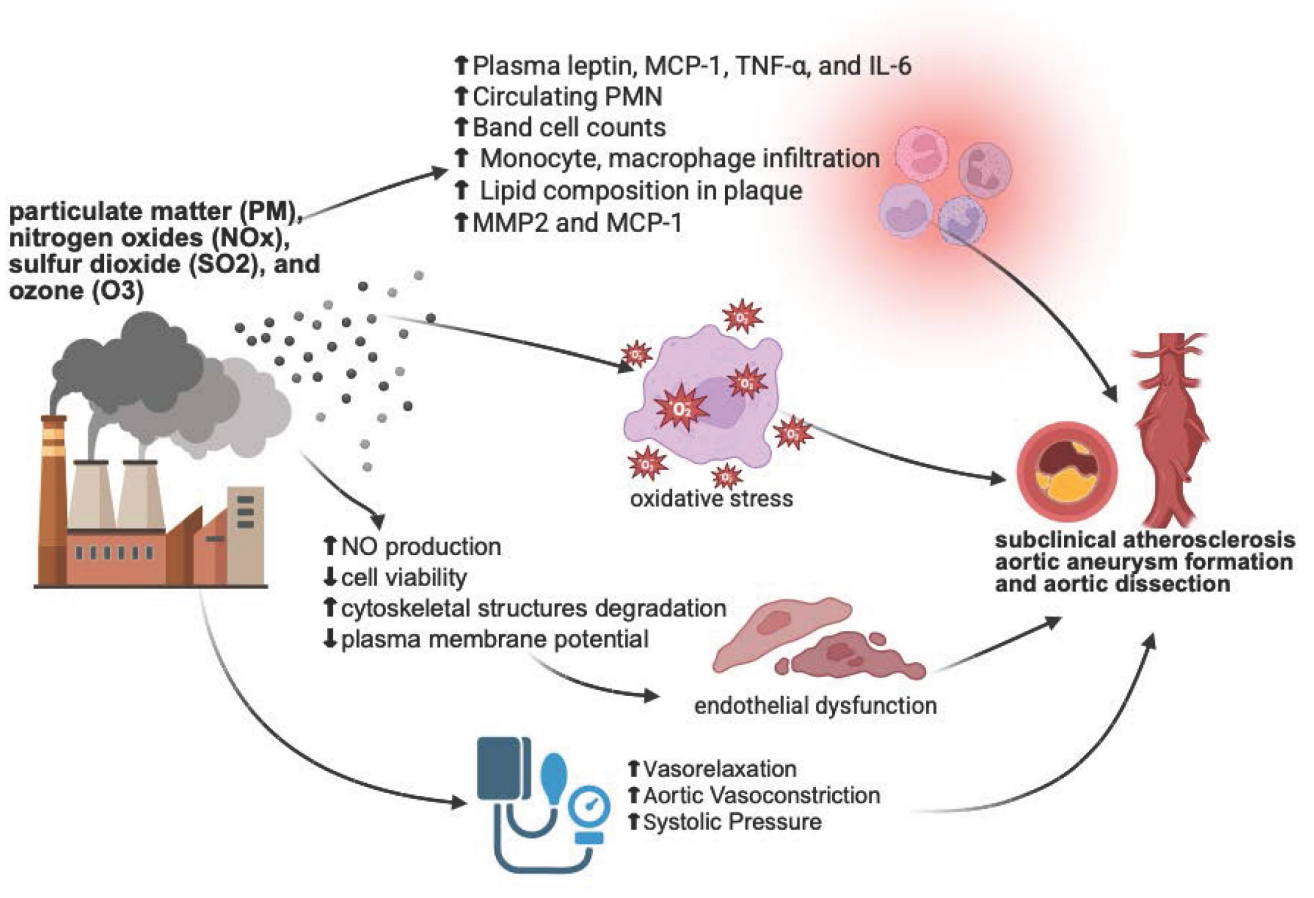
This figure illustrates the potential mechanisms by which air pollutants contribute to cardiovascular diseases, including subclinical atherosclerosis, aortic aneurysm formation, and aortic dissection. The inflammatory response is characterized by elevated levels of plasma leptin, MCP-1, TNF-α, IL-6, circulating polymorphonuclear neutrophils (PMN), band cell counts, monocyte and macrophage infiltration, lipid composition in plaques, MMP2, and MCP-1. Pollutants generate reactive oxygen species (ROS), leading to oxidative stress. Pollutant-induced endothelial dysfunction is marked by a decrease in nitric oxide (NO) production, cell viability, and plasma membrane potential, along with cytoskeletal degradation. Additionally, changes in blood vessel contractility result in increased systolic blood pressure. These mechanisms collectively contribute to the development of vascular diseases. The figure created with BioRender.

The evidence presented highlights and underscores the significant impact of air pollution on aortic disease. Particulate matter, especially PM_2.5_ and DEP play a crucial role in exacerbating conditions such as atherosclerosis, aortic aneurysms, and dissections. *In vivo* and* in vitro* studies reveal that oxidative stress, inflammation, and endothelial dysfunction are key mechanisms through which pollutants damage the aorta. Clinical studies further suggest strong associations between air pollution exposure and increased aortic disease risk, though findings vary across different populations and study designs. This information is summarized in **Figure 1.** Future research should emphasize preventive strategies, including the exploration of modifiable factors such as diet and nutrition, and translation of mechanistic insights into clinical applications. Comprehensive strategies integrating environmental monitoring, epidemiological research, basic mechanistic studies, and clinical applications are essential to address the growing cardiovascular health challenges posed by air pollution.

## Figures and Tables

**Table 1A:** Air Pollutants induce aortic atherosclerosis and aortic aneurysm: Evidence from *in vivo* studies. PM 2.5.

Model animal/age/sex	Type of air pollutants concentration/expose time / Route	Major findings				Interpretation	Ref
		Anatomical or histological change	Physiological change	Inflammation	Others		
C57BL/6J mice / 12 weeks old / Male	N/A / 9 days/ inhalation	-	-	↑ urinary excretion of acrolein metabolite (3HPMA) ↑ upregulated PVAT leptin mRNA expression	↑ protein-acrolein adducts in PVAT aorta	PM 2.5 attributed to the induction of chronic inflammation and oxidative stress in PVAT aorta.	29
Ang II-infused ApoE^-/-^ mice / eight weeks old / N/A	1 mg/ml / twice a week for 2-3 weeks/ intranasal	↑ AAA formation in angiotensin Ⅱ induced mice ↑expression of MMP2 and MCP-1	-	-	-	PM 2.5 promoted the formation of AAA in an Ang Ⅱ-induced AAA model.	6
C57BL/6 J mice with aortic aneurysm induced by BAPN /3-weeks-old/ Male	DEP1 mg/mL /Twice a week 2-6 weeks /Tracheal instillation	↑ aortic dissection in the BAPN mice ⟷diameters of the thoracic aorta ↑ apoptosis in the aortas of the exposed group	-	-	-	DEP exposure increased the incidence of AD in BAPN-induced TAAand increased apoptosis in the aorta.	9
HFD C57BL/6J ApoE^-/-^ mice /6-week-old/male	84.27 ± _28.84 μg/m3/ 3 months/inhalation After 1 month, mice received intraperitoneal injection of - Wnt5a inhibitor Box5 1 mg/kg Box5) - Ror2 inhibitor β-Arrestin2 1 mg/kg	↑size of the atherosclerotic plaques ↑lipid deposition in atherosclerotic plaques ↑IL-6, TNF-α, MCP-1, and leptin in PVAT	-	↑plasma leptin, MCP-1, TNF-α, and IL-6,	↓atherosclerotic lesion area by Wnt5a inhibitor Box5 or the Ror2 inhibitor β-Arrestin2 ↑plasma levels of LDL-C, TC, and TG	Fine particulate air pollution exposure promotes atherosclerosis through the inflammation process, and the Wnt5a inhibitor Box5 or the Ror2 inhibitor β-Arrestin2 attenuated this effect.	7
Tie2 GFP and Tie2 GFP/ ApoE^-/-^ mice /N/A /Male	300 and 1000 μg PM/m3 /Performed 4 h/day, 7 days/week for 4 weeks/Inhalation	-	-	-	↑genes dysregulated by >2.8-fold in the endothelium	Diesel exhaust impaired endothelial gene regulation in hyperlipidemic mice.	30
ApoE^-/-^ or LDLR-/- mice /8 weeks old/male	9.1 ± 7.3 μg/m3 /6 months/inhalation	↑lesion area, lipid and collagen content of atherosclerotic plaque in the aorta	-	-	↑7-ketocholesterol (7-KCh), ↑CD36 expression in the monocytes and macrophages	PM 2.5 induced atherosclerosis progression via CD36-mediated abnormal accumulations of 7-KCh.	8
Offspring from pregnant apolipoprotein E-deficient Mice/16 weeks old/ N/A	DE 250-300 lg/m3 PM 2.5 /6 h per day for 5 days per week from the determined time of mating until birth (mouse gestation period is approximately 19-21 days)/inhalation	⟷ average size or composition of early atherosclerotic lesions in either the aortic sinus	-	-	⟷total plasma cholesterol or triglyceride levels	In utero DE exposure did not affect lipoprotein metabolism, or risk of developing larger atherosclerotic lesions.	31
C57BL6 mice/ 8-wk-old/ Male/ isolated aorta	concentrated PM 2.5 / 6 h/day for 9 days Followed by *ex vivo* in isolated aortas stimulated with 100 nM of insulin	-	-	-	↓ Akt phosphorylation induced by insulin	PM 2.5 induced vascular insulin resistance.	32

**Table 1B:** Air Pollutants induce aortic atherosclerosis and aortic aneurysm: Evidence from *in vivo* studies; PM 10

Model animal/age/sex	Type of air pollutants concentration/expose time / Route	Major findings				Interpretation	Ref
		Anatomical or histological change	Physiological change	Inflammation	Others		
WHHL rabbits/ 42 weeks-old/ female	N/A / twice a week for 4 wk /intratracheal instillation	↑fraction of the aortic surface being taken up by atherosclerotic plaque ↑ICAM-1 and VCAM-1 expression on atherosclerotic plaques in the aorta ↑MoBrdU attachedonto the endothelium surface over plaquesand recruitment of MoBrdU into atherosclerotic plaques	-	↓ adhesion molecule expression of CD11b and CD18 on circulating monocytes	-	PM10 was associated with the induction of atherosclerosis by promoting the recruitment of circulating monocytes into atherosclerotic plaques.	10
WHHL rabbits/42 weeks old/ female	5 mg EHC-93 mixed with 1 ml of Saline/twice a week for four weeks/intra-pharyngeal instillation	↑volume fraction of the lesion taken up by extracellular lipids, total lipids in aortic wall	-	↑circulating PMN band cell counts ↑size of the bone marrow mitotic pool of PMNs	-	PM10 exposure induced systemic inflammatory response and accelerated atherosclerosis in the aorta.	11

**Table 1C:** Air Pollutants induce aortic atherosclerosis and aortic aneurysm: Evidence from *in vivo* studies; Other particles.

Model animal/age/sex	Type of air pollutants concentration/expose time / Route	Major findings				Interpretation	Ref
		Anatomical or histological change	Physiological change	Inflammation	Others		
Wistar Kyoto rats (WKY) / 13-15 weeks old/ Male/ Aortic Rings	EHC-93 particle / directly exposed/ 10-100 μg/ml	-	⟷contraction in isolated vessels↑ acute vasorelaxation	-	-	Ambient PM resulted in an acute vasorelaxation.	33
Male Wistar rats/ N/A / male/ thoracic aortas	BaP 20 mg/kg / weekly up to 8 weeks/intraperitoneal injection	-	↑SBP ↑phenylephrine-induced contractile response in endothelium-denuded aortas	-	↓BaP-enhanced vasoconstriction by NAC 2 mM, PKC inhibitor, MAPK inhibitor, and Rho-kinase inhibitor	BaP enhanced aortic vasoconstriction via the activation of ROS and muscular signaling molecules*.*	34
ApoE^-/-^ mice / N/A / male	- DE 996 (1.1) mg/m^3^ (% of measured mass)- GE 61 (50.1) mg/m^3^ (% of measured mass)- WS 1041 (5.3) mg/m3 (% of measured mass)- CE 1015 (37.3) mg/m3 (% of measured mass)/exposed for 6 h/day, 7 days/week for 50 days/inhalation	-	-	-	GE ↑ET-1, VEGF↑oxidative stress (HO-1, TBARS)↑ MMP-7 and 9, TIMP-2DE ↑ET-1, TIMP-2 ↑oxidative stress (TBARS)WS, CE no significant change in all parameters	GE and DE induced oxidative stress and pro-atheroscleroticresponses in the aorta.	12
ApoE^-/-^ mice/N/A /N/A	DEP 35 μL DEP (1 mg/mL, SRM-2975)/ twice weekly for 4 weeks/ oropharyngeal aspiration	↑lipid-rich lesions on the surface of the thoracic aorta ↑developed large lesions throughout the length of the brachiocephalic artery ⟷ plaque composition; foam cells, lipid cavities, smooth muscle cell collagen	⟷Phenylephrine concentration dependent contraction ⟷Relaxations to vasodilator; acetylcholine, NO	⟷C-reactive protein (CRP) and plasma fibrinogen ⟷MMP-2 and MMP-9	↑Serum cholesterol concentrations ⟷serum triglycerides	Exposure to diesel exhaust increased atherosclerotic plaques.	13
ApoE^-/-^ /10 weeks old/ male	DEP/ 100, 300, or 1,000 μg PM/m3 /6hrs/d 7d/wk for 50 days /inhalation	↑Macrophage staining, Collagen staining ⟷plaque area	-	-	↑MMP-9 ↓MMP13 ↑upregulation for endothelin-1, TIMP2 ↑lipid peroxides	DEP induced alterations in gene markers of vascular remodeling and aortic lipid peroxidation.	14
ApoE^-/-^ mice/ 10-12 weeks old/male	α-pinene-derived secondary organic aerosol (SOA)/ 200 μg m-3 PM/ inhalation/ 7 days	-	-	↑heme oxygenase-1, MMP-9 in mouse aorta	↑thiobarbituric acid-reactive substances (TBARS)	Short-term inhalation of SOA induced vascular inflammatory response via increased expression of HO-1, MMP-9, and TBARS in the aorta.	15

Abbreviations: AAA, Abdominal Aortic Aneurysm; AD, Aortic Dissection; BAPN, β-Aminopropionitrile; BaP, Benzo[15]pyrene; CAP, Concentrated Ambient PM 2.5; CASP, Central Aortic Systolic Pressure; CE, Coal Combustion Emissions; DE, Diesel Exhausts; DEP, Diesel Exhaust Particulate; GE, Gasoline Exhausts; HDL, High Density Lipoprotein; HCD, High Cholesterol Diet; HFD, High Fat Diet; LDL, Low Density Lipoprotein; LDLR, Low Density Lipoprotein Receptor; MAPK, Mitogen-Activated Protein Kinases; MoBrdU, BrdU-Labeled Monocytes; NLRP3, Nucleotide Binding Oligomerization Domain-Like Receptor Protein 3; PM, Particulate Matters; PMN, Polymorphonuclear Leukocytes; PVAT, Perivascular Adipose Tissue; ROS, Reactive Oxygen Species; TAA, Thoracic Aortic Aneurysm; TF, Tissue Factor; WS, Hardwood Smoke.

**Table 2A:** Air pollutants induce aortic atherosclerosis and aortic aneurysm: Evidence from *in vivo* studies through the comparison of the manipulation of genetic and/or dietary factors; PM 2.5.

Model animal/age/sex	Type of air pollutants concentration/expose time / Route	Major findings				Interpretation	Ref
		Anatomical or histological change	Physiological change	Inflammation	Others		
ApoE^-/-^ mice/ Six-week-old/ male			-	-	-	Exposure to PM 2.5 exaggerates atherosclerosis and TF expression.	16
ND+FA HFD +FA		plaque area from ultrasound image at aortic arch HFD+PM >ND+PM					
ND+PM HFD+PM	85 μg/m3 / 6h/day, 5 days/wk, for 6 mo / inhalation	TF in the plaques HFD+PM >ND+PM macrophage infiltration in the plaques HFD+PM >ND+PM					
ApoE^-/-^ C57BL/6 J mice /7-8weeks old/ Male	Different dose/ once a week for 8 weeks/ intratracheal instillation		-	-	-	Coal-fired PM 2.5 induced atherosclerosis in hyperlipidemic mice without a dose-dependent effect.	35
Normal control HFD control group		Histologic change HFD; Intima thickening			Atherosclerosis related protein ET-1 PM group>HFD control group		
PM group	- Low dose; 0.05 mg/kg of body weight 36/week - Middle dose; 0.50 mg/kg of bw/week - High dose; 5.00 mg/kg of bw/week	All PM treatment; intimal thickening, fibrous cap formation, and increased foam cells in the aorta			E-selectin expression PM group>HFD control >normal control		
ApoE^-/-^ mice/ six-week-old /male ND +PM HFD+PM ND +FA HFD+FA	PM 2.5 235.76±72.73 μg/m3 in PM chamber 10.22±2.53 μg/m3 in FA chamber 8 h per day, 7 days per week for 16 weeks / inhalation	Aortic root plaque area ND+PM > ND+FA HFD+PM>HFD+FA	-	CD 36 in serum and aorta, IL-1β and IL-18 ND+PM > ND+FA HFD+PM>HFD+FA HFD+FA>ND+FA HFD+PM>ND+PM NLRP3 inflammasome-related proteins in the aorta ND+PM > ND+FA HFD+PM>HFD+FA	Apo B, T-CHO and TG ND+PM > ND+FA HFD+PM>HFD+FA LDL-C HFD+PM>HFD+FA Apo A1 and HDL-C ND+PM < ND+FA HFD+PM<HFD+FA	PM 2.5-induced development and progression of atherosclerosis via increased inflammation and altered serum lipid.	17
ApoE^-/-^ mice/ Six-weeks old/ male					-	PM 2.5 altered vasomotor toneand induced vascular inflammation.	5
ND+FA HFD +FA		plaque area HFD+PM> HFD+FA ND+PM= ND+FA	vasoconstrictor responses to phenylephrine and serotonin challenge in the thoracic aorta HFD+PM> HFD+FA	inducible isoform of nitric oxide and protein nitration product 3-nitrotyrosine HFD+PM> HFD+FA ND+PM> ND+FA			
ND+PM HFD+PM	PM 2.5 85 μg/m3 /6 hours per day, 5 days perweek for a total of 6 months/ inhalation		relaxation to the endothelium-dependent agonist acetylcholine HFD+PM>HFD+FA				

**Table 2B:** Air pollutants induce aortic atherosclerosis and aortic aneurysm: Evidence from *in vivo* studies through the comparison of the manipulation of genetic and/or dietary factors; Others particles/ mixed particles.

Model animal/age/sex	Type of air pollutants concentration/expose time / Route				Major findings				Interpretation	Ref
					Anatomical or histological change	Physiological change	Inflammation	Others		
C57BL/6 LDLR^-/-^ mice /Four weeks old /male						-	-		Air pollution influenced atherosclerosis plaque thickness especially in hyperlipidemic mice.	18
NF-HCD NF-ND	PM 2.5 34.6 μm/m3 PM 10 34.6 μm/m3 black smoke 45.5 μm/m3NO2 70.5 μm/m3 /24 h/day, seven days a week for four months /Inhalation				thickness of the atherosclerosisthe arterial wall NF-HCD>FA-HCD> others group			LDL plasma oxidation, anti-oxLDL and anti-apoB-D NF > FA		
FA-HCD FA-ND	PM 2.5 1.63 μm/m3 PM 10 2.68 μm/m3 black smoke 2.18 μm/m3NO2 60.33 μm/m3 /24 h/day, seven days a week for four months /Inhalation									
C57BL/6J ApoE^-/-^ and ApoE^+/+^ mice / 11-13 weeks old /Female	DEP suspended in saline/0, 0.5 and 5 mg/kg bodyweight. /Injection 1hr prior to sacrificed					Endothelium-dependent vasorelaxation EC50 value, Emax ApoE^-/-^ mice + 0.5 mg/kg > ApoE^+/+^ +0 mg/kg		-	Exposure to DEP had an acute effect on vasorelaxation.	27
homozygous C57BL/6J LDL receptor knockout (LDLR KO) mice /4-6-weeks old /male						-	-		PM-induced atherosclerosis, and NAC attenuated the PM-potentiated atherosclerosis.	19
HFD HFD + NAC					Atherosclerotic plaque formation PM+HFD>HFD>PM+HFD+NAC>HFD+NAC			6 months PM Blood ox-LDL PM+HFD>HFD>PM+HFD+NAC>HFD+NAC		
PM + ND PM + HFD PM + HFD + NAC	10 μg of PM (mean particle diameter, < 4 μm)/three times/week for 1 week or 6 months/intranasal instillation									
										

Abbreviations: FA, Filtered Air; HDL, High Density Lipoprotein; HCD, High Cholesterol Diet; HFD, High Fat Diet; LDL, Low Density Lipoprotein; LDLR, Low Density Lipoprotein Receptor; NLRP3, Nucleotide Binding Oligomerization Domain-Like Receptor Protein 3; ND, Normal Diet; NF, Non-Filtered Air; PM, Particulate Matters; PMN, Polymorphonuclear Leukocytes; TF, Tissue Factor.

**Table 3:** Air Pollutants Induce aortic disease: Evidence from *in vitro* studies.

Cell	Type of air pollutants concentration/expose time	Related mechanism	Interpretation	Ref
Human aortic endothelial cells	UFP 50μg/mL /6 hours	↓ endothelial NO production via eNOS S-glutathionylation	UFP reduced vascular endothelial NO production	20
Human Aortic Endothelial Cells	DEPs 10 mg/ml 50 mg/ml, 100 mg/ml /Four exposure times;4, 8, 24, and 48 hours	↓in cell viability ↑cytoskeletal structures degradation ↓plasma membrane potential (PMP)	DEPs could negatively impair cell viability and alter membrane nanostructures and cytoskeleton components	21
Macrophages RAW264.7	Aqueous PM 2.5 extract	↑ lipid accumulation, ↑ classical macrophage (M1) activation, ↑ HSP70 expression	Aqueous PM 2.5 promotes pro-inflammatory macrophage polarization and stress response, contributing to vascular inflammation and atherogenesis	22
Macrophages	PM 2.5	NLRP3 inflammasome activation → ↑ IL-1β production (via oxidative stress, mitochondrial ROS, lysosomal damage)	PM 2.5 primes macrophages to trigger NLRP3-mediated inflammatory responses, highlighting a key pathway linking particulate exposure to vascular injury	23
Endothelial cells, macrophages	Aqueous PM 2.5 extract	Oxidative stress, loss of barrier function inflammatory cytokine release	PM 2.5 induces endothelial dysfunction both directly and indirectly via macrophage activation	37

Abbreviations: DEP, Diesel Exhaust Particulate; HSP, heat-shock response; UFP, Ultrafine Particles; NO, nitric oxide; NLRP3, NOD-like receptor protein 3 inflammasome; ROS, reactive oxygen species.

**Table 4:** Air Pollutants Induce aortic disease: Evidence from clinical studies

Subjects/Age (yrs)/ Country	Type of Study	N	Type of air pollutants			major findings		Interpretation	Ref
			Concentration/expose time						
			PM 2.5	PM 10	others	Image finding	Hemodynamic		
adult/35-75 years/Beijing, China	prospective panel study	65	PM2.5 99.5 mg/m3 /N/A	PM10 121.3 mg/m3/N/A	black carbon, 6.5 mg/m3 sulfur dioxide, 24.5 mg/m3nitrogen dioxide was 59.2 mg/m3 /N/A	-	↑ Each 10 mg/m3 at MA28 increase CASPPM10 0.87 mm Hg (95% CI: 0.42, 1.32)PM2.5 0.81 mm Hg (95% CI: 0.24, 1.38)SO2 2.01 mm Hg (95% CI: 0.32, 3.71)	PM10, PM2.5 associated with increased central aortic systolic pressure	24
adult/45-75 years/Germany	prospective cohort	4238	-	-	-	↑TAC-burden of 18.1% (95% CI: 6.6; 30.9%) per 2.4 mg/m3 PM2.5	-	PM2.5 increased TAC	25
adult/45-84 years/5 U.S. metropolitan areas	retrospective cross-sectional study	1147	15 μg/m3 /N/A	-	-	⟷ aortic calcification	-	PM2.5 not associated with abdominal aortic calcification	26
adults/45-75 years/Germany	retrospective cohort	3155	16.7(1.2) mg/m3 /N/A	20.2 (2.6) mg/m3 /N/A	NO2 39.4(4.0) mg/m3 /N/A	⟷ development and progression of TAC	-	no association between traffic related air pollution and development and progression of subclinical atherosclerosis	36
adult/age>35 years (men) or >40 years (women)/ Northeastern USA.	prospective cohort	3506	-	-	-	⟷presence or extent of TAC or AAC, or with AAC progression	-	no association between PM2.5 and the presence or extent of TAC or AAC, or with AAC	28

Abbreviations: AAC, Abdominal Aortic Calcium; CASP, Central Aortic Systolic Pressure; MA, moving average; CASP, Central Aortic Systolic Pressure; TAC, Thoracic Aortic Calcification.
